# In vitro thermotherapy-based methods for plant virus eradication

**DOI:** 10.1186/s13007-018-0355-y

**Published:** 2018-10-06

**Authors:** Min-Rui Wang, Zhen-Hua Cui, Jing-Wei Li, Xin-Yi Hao, Lei Zhao, Qiao-Chun Wang

**Affiliations:** 1 State Key Laboratory of Crop Stress Biology for Arid Areas, College of Horticulture, Northwest A&F University, Yangling, 712100 Shaanxi China; 2 College of Horticulture, Qingdao Agriculture University, Qingdao, 266109 Shandong China; 3 State Key Laboratory of Crop Stress Biology for Arid Areas, College of Plant Protection, Northwest A&F University, Yangling, 712100 Shaanxi China

**Keywords:** Cryotherapy, Chemotherapy, Micrografting, Shoot tip culture, Thermotherapy, Virus eradication

## Abstract

Production of virus-free plants is necessary to control viral diseases, import novel cultivars from other countries, exchange breeding materials between countries or regions and preserve plant germplasm. In vitro techniques represent the most successful approaches for virus eradication. In vitro thermotherapy-based methods, including combining thermotherapy with shoot tip culture, chemotherapy, micrografting or shoot tip cryotherapy, have been successfully established for efficient eradication of various viruses from almost all of the most economically important crops. The present study reviewed recent advances in in vitro thermotherapy-based methods for virus eradication since the twenty-first century. Mechanisms as to why thermotherapy-based methods could efficiently eradicate viruses were discussed. Finally, future prospects were proposed to direct further studies.

## Necessity to produce virus-free plants

Virus diseases cause great losses of crop yield and have long been a constraint for sustainable developments of agricultural production [1, 2]. For example, potato leafroll virus (PLRV), potato virus S (PVS), potato virus X and potato virus Y (PVY) are among the most serious viruses attacking potato [3]. Single infection caused yield losses of 40–60% by PLRV, 10–20% by PVS, 10–50% by PVX and 20–50% by PVY [4]. Mixed infection with two viruses resulted in a much larger loss of yield than the single infection [4]. Plum pox virus (PPV), one of the most serious viral diseases attacking *Prunus* fruit trees, widely occurred in almost all stone fruit producing countries [5]. Annual yield losses caused by PPV infection were 1.5 million for plum and 0.6 million metric tons for apricot, approximately valuing at €5400 million and €3600 million for the former and latter in Europe [5]. By 2013, over €33 million had been invested in research projects on PPV control in Europe [5].

Plant viruses are obligate intracellular parasites that colonize only inside the living cells of the host and can be transmitted by vegetative propagation from generation to generation and insect vectors from the virus-infected plants to the healthy ones [6]. Although the use of chemicals had potential applications to control viral diseases [7, 8], cultivation of virus-free plants has been/is an agricultural strategy for efficient control of them [2, 9]. As early as 1968, the European Union (EU) issued an EU Council directive [10], which required that propagative materials of fruit crops must meet the phytosanitary requirements. Virus-free plants are currently widely grown throughout the world to control viral diseases in many of economically important crops like tuber crops [4, 11], fruit trees [2, 12], herbaceous ornamentals [13, 14]. Virus-free materials are required in importing novel cultivars from other countries and exchanging breeding materials between countries or regions [2, 15]. In addition, preservation of plant germplasm also emphasizes use of virus-free plants [16, 17].

**Fig. 1:**
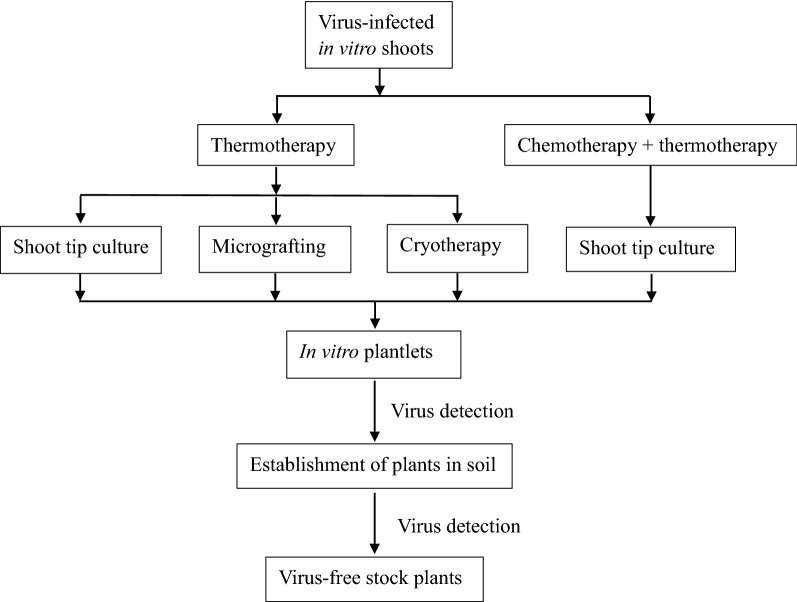
I*n vitro* thermotherapy-based methods for production of virus-free plants

In vitro culture techniques represent the most successful strategies for production of virus-free plants [18–20]. So far, various methods have been established for eradication of plant viruses, including shoot tip culture (also called meristem culture) [2, 4, 11, 12, 19, 21, 23], micrografting [12, 21], chemotherapy [12, 17, 21], thermotherapy [2, 11, 19, 22, 23] and shoot tip cryotherapy [24, 25]. Accumulative data have proven combining thermotherapy with each of them (herein called thermotherapy-based methods, Fig. 1) are much more efficient for virus eradication than single use of them. For example, shoot tip cryotherapy completely failed to eradicate raspberry bushy dwarf virus (RBDV) [26] and apple stem grooving virus (ASGV) [27], while combining thermotherapy with shoot tip cryotherapy produced 33% and 100% of plants free of RBDV [22] and ASGV [26].

Detailed information on the said subject in the last century can be found in a number of excellent reviews [2, 12, 19, 21–23]. The present study reviewed recent advances in thermotherapy-based methods for virus eradication since the 21st century. Mechanisms as to why thermotherapy-based methods could efficiently eradicate virus eradication were discussed. Finally, future prospects were also proposed to direct further studies.

## Thermotherapy-based methods for virus eradication

In thermotherapy-based methods, infected in vitro cultures are first heat-treated and then subjected to a given procedure (Fig. 1), as described below.

In general, the higher the temperature and the longer the exposure duration are, the higher the virus-eradication frequency is. Often, thermotherapy of 35–42 °C for 4–6 weeks is applied to the target plants, mainly depending on virus type and plant species, as well as the virus-host combination [2, 12, 19, 21–23]. Choice of a thermotherapy regime should allow the treated plant to survive and at the same time inactivate the virus, thus resulting in production of virus-free plants.

### Combining thermotherapy with shoot tip culture

This technique includes thermotherapy of the diseased in vitro shoots, followed by shoot tip culture.

Skiada et al. [28] reported combining thermotherapy with shoot tip culture for eradication of grapevine leafroll-associated virus 1 (GLRaV-1), an easy-to-eradicate virus, and grapevine rupestris stem pitting-associated virus (GRSPaV), a difficult-to-eradicate virus, from grapevine ‘Agiorgitiko’. The in vitro shoots mix-infected with GLRaV-1 and GRSPaV were heat-treated for 6 weeks by an alternating temperature of 40 °C/37 °C (day/night), followed by shoot tip culture. This procedure resulted in about 53% survival of the heat-treated shoots, 56% regeneration of shoot tips, and 91% and 74% of GLRaV-1- and GRSPaV-1-free plants. Thermotherapy followed by shoot tip culture was reported to eradicate PVY from infected potato [29]. In vitro virus-infected potato shoots were thermo-treated for 40 days at a consistent temperature of 37 °C, followed by shoot tip culture (0.1–0.3 mm). Culture of 0.1 mm shoot tips resulted in about 88% survival levels of the treated shoots and 75–81% virus-free plants in two potato cultivars, and larger shoot tips decreased the virus eradication frequencies [29].

Tobacco mosaic virus (TMV) represented a type of viruses that were still difficult to eradicate by thermotherapy followed by shoot tip culture [30]. Greenhouse-grown plants of soybean (*Glycine max*) were mechanically inoculated with tobacco mosaic virus and then thermo-treated at 40 °C/6 °C (day/night) for 15 days. Heat-treated plants grew as well as the control (25 °C/6 °C). Virus infectivity increased in the leaves of the treated plants heat-treated in these two thermotherapy regimes. TMV was detected in all the newly developed news. Shoot tips that developed during the alternating temperature treatments were isolated from the treated TMV-infected tobacco and cultured in vitro for plantlet regeneration. As results, all plantlets regenerated were still TMV-infected [30].

Thermotherapy followed by shoot tip culture was the most frequently used method for virus eradication from plants including herbaceous crops and woody species. Some examples of successful virus eradication by thermotherapy followed by shoot tip culture are listed in Table 1.

**Table 1:** Some examples of in vitro thermotherapy followed by shoot tip culture for virus eradication

Plant species and cultivars or genotypes	Viruses	Temperature and duration	Shoot survival after thermotherapy (%)	Size of shoot tips (mm)	Shoot tip survival (%)	Virus-free frequency (%)	References
*Herbaceous crops*							
Potato ‘Diamond’	Potato virus Y (PVY)	37 °C, 40 days	43	1.0 cm	NT	33	[64]
Potato ‘Burren’ and ‘Binella’	PVY	37 °C, 40 days	NT	0.1–0.3	88–100	56–81	[25]
Potato genotype ‘040138’	Potato virus X (PVX)	32 °C/42 °C, (day/night), 35 days	80–100	NS	NT	80	[66]
Cassava ‘Olho Junto’	Cassava common mosaic virus (CsCMV)	39 °C/28 °C, (day/night), 30 days	NT	NS	NT	72	[115]
Cassava ‘Kibandameno’	East African cassava mosaic virus (EACMV)	35 °C, 42 days	81	NS	NT	78	[55]
Garlic ‘Morado’, ‘Union’, ‘Fuego’, ‘Lican’, ‘Nieve’, ‘Castaño’, ‘Violeta’, ‘Gostoso’, ‘Norteño’, ‘Perla’, ‘Sureño’	OYDV, LYSV, GCLV, garlic mite-borne filamentous virus (GarMbFV), garlic virus D (GarVD)	36 °C, 30–40 days	55–57	0.3	NT	63–100 (OYDV), 80–100 (LYSV), 93–100 (GCLV), 67–100 (GarMbFV), 18–97 (GarVD)	[116]
Garlic (cultivars not specified)	Onion yellow dwarf virus (OYDV) and leek yellow stripe virus (LYSV)	50 °C, 2 h	NT	0.3–0.5	NT	19 (OYDV) 33 (LYSV)	[117]
Garlic ‘Hamedan’	OYDV and garlic virus A, B, C, D (GarVs)	36 °C, 5 weeks	NT	1.0–2.0	71	0 (OYDV) 90 (GarVs)	[118]
Garlic ‘Jonas’	OYDV, LYSV and garlic common latent virus (GCLV)	38 °C, 30 days	NT	0.3–0.5	72	83 (OYDV) 100 (LYSV) 39 (GCLV)	[112]
*Herbaceous crops*							
Chrysanthemum ‘Regol Time’	Chrysanthemum B virus (CVB)	38 °C, 30 days	NT	0.3–1.0	NT	12	[28]
*Lilium *×* elegans* genotypes 409 and 599	Lilium symptomless virus (LSV)	35 °C, 42 days	NT	0.3	NT	100	[83]
*Lilium* Asiatic hybrid ‘Visconti’, and LA hybrids ‘Fangio’ and ‘Lacorno’	LSV, lily mottle virus (LMoV) and cucumber mosaic virus (CMV)	35 °C, 5 weeks	NT	NS	NT	100 (LSV) 100 (CMV) 100 (LMoV)	[51]
Begonia	Prunus necrotic ringspot virus (PNRSV)	38 °C/22 °C, (day/night), 35 days	NT	2–3 cm	NT	37.5	[63]
Horseradish	Turnip Mosaic Virus (TuMV)	37 °C, 23 days	100	0.5	NT	100	[53]
Artichoke	Artichoke Italian latent virus (AILV), artichoke latent virus (ArLV)	38 °C, 15 days	NT	0.3–0.8	75	100	[84]
*Woody species*							
Apple ‘Idared’ and ‘Sampion’	Apple chlorotic leaf spot virus (ACLSV) and (ASPV) (Idared) ACLSV, ASPV and apple stem grooving virus (ASGV) (Sampion)	39 °C, 6 days	NT	1.0–2.0	63 (Idared) 44 (Sampion)	60 (Idared) 0 (Sampion)	[71]
Apple ‘Yanfu9’, ‘Xinyanfu3’, ‘Xin2001’, ‘Gala’, Huafu, ‘Apple 123’ and ‘Zhengzhou No. 5’	ACLSV, ASGV, ASPV and apple mosaic virus (ApMV)	38 °C, 30 days	NT	1.0	26 (Total)	43 (Total)	[40]
Woody species							
Apple (*Malus *×* domestica* cvs. Gala, Ruiyang, Nongguo 25, Fuji, *M. paradisiaca* M9)	ASGV	36 °C/32 °C, (day/night), 4 weeks	100	1.5	63–90	7–38	[23]
Apple ‘Oregon Spur-II’	ACLSV, ApMV, ASGV, ASPV	37–40 °C, 4 weeks	NT	0.3–0.6	26–33	75 (ACLSV) 100 (ApMV, ASGV and ASPV)	[77]
Pear ‘Huang-hua’	ASGV and ACLSV	37 °C, 35 days	64	1.0	NT	66.7 (ACLSV) 33.3 (ASGV)	[60]
Pear ‘Fengshui’, ‘Jingshui No. 1’, ‘4’ and ‘14’	ASGV, ACLSV and ASPV	42 °C/34 °C, (day/night), 55 days	20–57	0.5–1.0	NT	100	[58]
Pear ‘Jinshui no. 2’	ASGV ACLSV	35 °C, 40 days	100	0.5	66.7	33.3	[61]
Apricot ‘Bebecou’	Plum pox virus (PPV)	35–37 °C, 20 days	NT	1.0–2.0 (meristem), 5.0 cm (Shoot tip)	35 (Meristem), 28 (Shoot tip)	74 (Meristem) 82 (Shoot tip)	[69]
Nectarine ‘Arm King’	PPV and prunus necrotic ringspot virus (PNRSV)	35 °C, 2 weeks	NT	1.3–2.0	38	86 (PPV) 81 (PNRSV)	[56]
*Woody species*							
Plum ‘Earliblue’	PNRSV	35–38 °C, 10–12 days	14–71	5.0	NT	100	[57]
Grapevine ‘Napoleon’	Grapevine leafroll-asso ciated virus-3 (GLRaV-3), grapevine fan leaf virus (GFLV)	37 °C/34 °C, (day/night) 1.5 months	NT	0.5–3.0	53–80	72–100 (GLRaV-3) 100 (GFLV)	[79]
Grapevine ‘Sagrantino’	Grape virus A (GVA)	36 °C, 57 days	NT	1.0 cm	NT	60	[72]
Grapevine ‘Mantilaria’ and ‘Prevezaniko’	Grapevine leafroll associated virus (GLRaV) and GRSPaV	40 °C/37 °C, (day/night), 1 weeks	59.37 (Mantilaria) 41.86 (Prevezaniko)	5.0	96 (Mantilaria) 78 (Prevezaniko)	38 (Mantilaria) 89 (Prevezaniko)	[81]
Grapevine ‘Bidaneh Sefid’ and ‘Shahroodi’	GFLV	40 °C/30 °C, (day/night), 7 weeks	NT	NT	NT	100	[119]
Grapevine ‘Agiorgitiko’	GLRaV-1 and GRSPaV	40 °C/37 °C, (day/night), 1 weeks	53	0.1–0.2 (Meristem) 5.0 (Shoot tip)	56 (Meristem) 88 (Shoot tip)	62 (Meristem) 38 (Shoot tip)	[24]
Grapevine *‘*Kober 5BB’	GVA, GFLV, grapevine fleck virus (GFkV), GLRaV-1 and GLRaV-3	37 °C, 48 days	100	NS	NT	100 (GFLV), 70 (GVA), 25 (GFLaV-1), 25 (GLRaV-3) and 0 (GFKV)	[86]
Fig ‘Biadi’ and ‘Aswad’	Fig leaf mottle-associated virus-1 and 2 (FLMaV-1 and FLMaV-2), and fig mosaic virus (FMV)	35 °C, 30 days	NT	6.0	39 (Biadi) 42 (Aswad)	0–81	[87]
Raspberry ‘Gatineau’	Raspberry bushy dwarf virus (RBDV)	37 °C, 21 days	NT	0.2–0.3	NT	39	[120]
Black raspberry (cultivars not specified)	Black raspberry necrosis virus (BRNV)	29 °C and 38 °C (in 4-h interval), 5 weeks	NT	NS	90	100	[62]
Caper (cultivars not specified)	Caper latent virus (CapLV)	38 °C, 45–60 days	97	0.3–0.4	60–93	89–93	[80]

*NS* not specified, *NT* not tested

### Combining chemotherapy with thermotherapy and shoot tip culture

In this technique, diseased in vitro shoots were cultured on an antivirus chemical-containing medium and then subjected to heat treatments, followed by shoot tip culture. In a few cases, diseased in vitro shoots were first heat-treated and then cultured on antivirus chemicals-containing medium, followed by shoot tip culture [31, 32]. Although several antivirus chemicals were available against plant viruses, ribavirin was the most frequently used, and sometimes 2-thiouracil was also used, for virus eradication [12, 19, 23]. Concentrations of antivirus agents used for virus eradication ranged between 20–50 mg L^−1^ for ribavirin [12, 19, 23] and 25–40 mg L^−1^ for 2-thiouracil [33, 34], depending on types of virus and hosts, as well as the virus-host combinations.

Fletcher and Fletcher [35] reported combining chemotherapy with thermotherapy for virus eradication from three Andean root crops including oca *(Oxalis tuberosa*), ulluco (*Ullucus tuberosus*) and arracacha (*Arracacia xanthorrhiz*). Diseased in vitro shoots were grown on a growth medium composed of MS supplemented with 50 mg L^−1^ ribavirin, which was added to the medium before autoclaving, and the cultures were then thermo-treated by an alternating temperature of 35 °C/31 °C (day/night). After about 10 days of thermotherapy, new shoots (1 cm in length) developed from the lateral shoot apices of oca and ulluco, and apical shoot apices of arracacha were excised and cultured for shoot regeneration on the same medium without ribavirin. This protocol produced about 80% of explant survivals in all three crops. Plants regenerated were free of arracacha virus B (AVB), papaya mosaic virus (PapMV) and ullucus mild mottle virus (UMMV) in 7 out of 8 accessions, but still infected with UMMV in one accession in oca. PapMV, ullucus virus C (UVC), UMMV and ullucus mosaic virus (UMV) were successfully eradicated in 5 accessions of ulluco, and arracacha virus A (AVA) was eradicated in arracacha ‘Racacha Blanca’.

Combining chemotherapy with thermotherapy was reported to eradicate apple viruses from diseased in vitro shoots [36]. Virus-infected in vitro apple shoots were cultured on a shoot maintenance medium composed of MS medium supplemented with 25 µg mL^−1^ ribavirin, which was filter-sterilized using a Millipore filter (0.22 µm) and added into the medium after autoclaving. The cultures were then thermo-treated at a constant temperature of 36 °C. After 20 days of thermotherapy, shoot tips (1.0 mm in size) were excised from the treated axillary shoots and cultured for shoot regeneration. About 90% of the treated shoots survived. All shoot tips regenerated into shoots, and all shoots regenerated were free of apple chlorotic leaf spot virus (ACLSV), apple stem pitting virus (ASPV) and ASGV.

Combining chemotherapy with thermotherapy was frequently used for virus eradication in herbaceous crops and sometimes also in woody plants. Some examples of virus eradication by combining chemotherapy with thermotherapy are listed in Table 2.

**Table 2:** Some examples of in vitro chemotherapy followed by thermotherapy for virus eradication

Plant species and cultivars or genotypes	Viruses	Antiviral chemicals (mg/L)	Survival of the treated shoots (%)	Survival of shoot tips (%)	Virus-free frequency (%)	References
*Herbaceous crops*						
Garlic (10 accessions)	OYDV, LYSV, GCLV, shallot latent virus (SLV) and MbFV	Ribavirin (50)	NT	NT	100 (OYDV, SLV and LYSV), 90 (GLCV) and 88 (MbFV)	[12]
Oca (8 accessions), ulluco (5 accessions), arracacha ‘Racacha Blanca’	Papaya mosaic virus (PapMV), arracacha virus B (AVB) and ullucus mild mottle virus (UMMV) in Oca; PapMV, ullucus virus *C* (UVC), UMMV, ullucus mosaic virus (UMV) in ulluco; arracacha A virus (AVA) in arracacha	Ribavirin (50)	≥ 80%	NT	(% not specified) free of viruses in 7 accessions, and still infected with UMMV in one accession in oca, free of viruses in 5 accessions in ulluco, and free of virus in arracacha	[30]
Potato ‘Baraka’	PVY	Ribavirin (20)	NT	NT	83	[74]
Potato ‘F9-99’	PLRV and PVY	Ribavirin (20)	45–85	NT	26–60 (PLRV) 98–100 (PVY)	[49]
Potato ‘Diamond’	PVY	Ribavirin (20)	26	NT	43	[64]
*Begonia *×* semperflorens*	PNRSV	Ribavirin (20)	NT	NT	58	[63]
Chrysanthemum ‘Regol Time’	Chrysanthemum B virus (CVB)	2-Thiouracil (40)	NT	NT	27	[28]
Chrysanthemum ‘Regol Time’	Tomato aspermy virus (TAV)	Ribavirin (10)	NT	NT	52	[91]
*Herbaceous crops*						
Cassava ‘Cidade Rica’, ‘Riqueza II’, ‘Mandioca Lagoa’, ‘CM-425/7’, ‘Sabará’, ‘Sauma’	Cassava frogskin disease (CFSD) and cassava vein mosaic virus (CsVMV)	Tetracycline (5–15)	100	NT	100 (CFSD) 98 (CsVMV)	[54]
*Woody plants*						
Apple ‘JonagoId’, pear ‘Pierre Corneille’, raspberry ‘Norna’	ACLSV and ASGV in apple, ACLSV in pear and raspberry vein chlorosis virus (RVCV) in raspberry	Ribavirin (50)	42 (apple), 80 (pear) and 34 (raspberry)	NT	100 (ACLSV and ASGV in apple), 83 (ACLSV in pear), 89 (RVCV in raspberry)	[71]
Apple ‘Xinhongjiangjun’	ASPV, ASGV and ACLSV	Ribavirin (25)	90	59 (apical shoots) and 100 (axillary shoots)	94 (apical shoots) 100 (axillary shoots)	[31]
Pear ‘Pierre Corneille’	ACLSV	Ribavirin (50)	71	NT	90	[26]
Pear ‘Jinshui no. 2’	ASGV, ACLSV	Ribavirin (25)	100	71	100	[61]

*NT* not tested

### Combining thermotherapy with micrografting

Micrografting is referred as the placement of a small meristem or a section of microshoot onto the top of a rootstock cultured in vitro [37]. Murashige et al. [38] and Navarro et al. [39] were the first to use micrografting for virus eradication from diseased citrus in vitro cultures. Since then, a number of studies on micrografting for virus eradication have been reported [12, 21, 37].

There have been only a few studies using thermotherapy followed by micrografting for virus eradication [40–42]. Several studies that used in vivo system were not included in the present study [43–46]. Sharma et al. [41] reported combining thermotherapy with micrografting for efficient eradication of *Indian citrus ringspot virus* (ICRSV) from the diseased plants of Kinnow (*Citrus nobilis *× · *Citrus deliciosa*). One-year-old pot-grown plants were exposed to 38–40 °C by gradually increasing temperatures (1 °C/day) from 30 °C to 38–40 °C within 9–11 days and then maintained at 38–40 °C until new shoots elongated. Shoot tips (0.7 mm) were excised from the new shoots, surface-disinfected and micrografted upon in vitro 2-weeks old seedling rootstocks of rough lemon (*C. jambhiri*). Micrografts developed into plantlets after 5–6 weeks of micrografting. This procedure resulted in 28–40% of micrografting success rates and 59–60% of ICRSV-free frequencies.

Applying thermotherapy (40 °C, 1 week) followed by micrografting, Chae et al. [42] completely eradicated citrus tristeza virus (CTV), satsuma dwarf virus (SDV) and citrus tatter leaf virus (CTLV) for six citrus cultivars.

Combining thermotherapy with micrografting was mainly applied to woody plants like citrus [38–43], apple [44–46] and pear [46], because shoot regeneration from shoot tip culture is difficult in these plants [12]. Some examples of successful eradication by combining thermotherapy with micrografting are listed in Table 3.

**Table 3:** Some examples of thermotherapy followed by in vitro micrografting or shoot tip cryotherapy for virus eradication

Methods	Plant species and cultivars or genotypes	Viruses	Size of shoot tips (mm)	Survival of the treated shoots (%)	Success (%) of micrografting or shoot regrowth (%) in cryotherapy	Virus-free frequency (%)	References
Thermotherapy + micrografting	Citrus ‘Early satsuma mandarin’, ‘Yuzu’, ‘Shiranuhi’	Citrus tristeza virus (CTV), satsuma dwarf virus (SDV), citrus tatter leaf virus (CTLV)	0.3	NT	13–75	76 (CTV), 100 (SDV) and 83 (CTLV)	[35]
	*Citrus* hybrid *‘*Kinnow’	Indian citrus ringspot virus (ICRSV)	0.7	42 (treatment of nodal segments at 50 °C for 120 min in water bath), 28 (exposure of nodal segments to 50 °C for 120 min of hot air) and NT (exposure of whole plants to 38 °C for 9 days in growth chamber)	38 (water bath), 26 (hot air) and 40 (growth chamber)	37 (water bath), 23 (hot air) and 59 (growth chamber)	[36]
	Citrus ‘Ehimekashi dai28go’, ‘Setoka’, ‘Shiranuhi’, ‘Haraejosaeng’, ‘Pungkwang’, ‘Samdajosaeng’	CTV	NS	NT	NT	100	[37]
Thermotherapy + cryotherapy	Raspberry genotype ‘Z13’	Raspberry bushy dwarf virus (RBDV)	1.0	NT	30	35	[22]
	Garlic ‘Jonas’	OYDV, SYSV and GCLV	NS	NT	40	90 (OYDV) 100 (LYSV) 80 (GCLV)	[112]
	Apple ‘Gala’, ‘Ruixue’, ‘Ruiyang’, ‘Nongguo 25’, ‘Fuji’and *‘*M9’	ASGV	1.5	100	33–76	30–100	[23]

*NT* not tested, *NS* not specified

### Combining thermotherapy with shoot tip cryotherapy

**Fig. 2:**
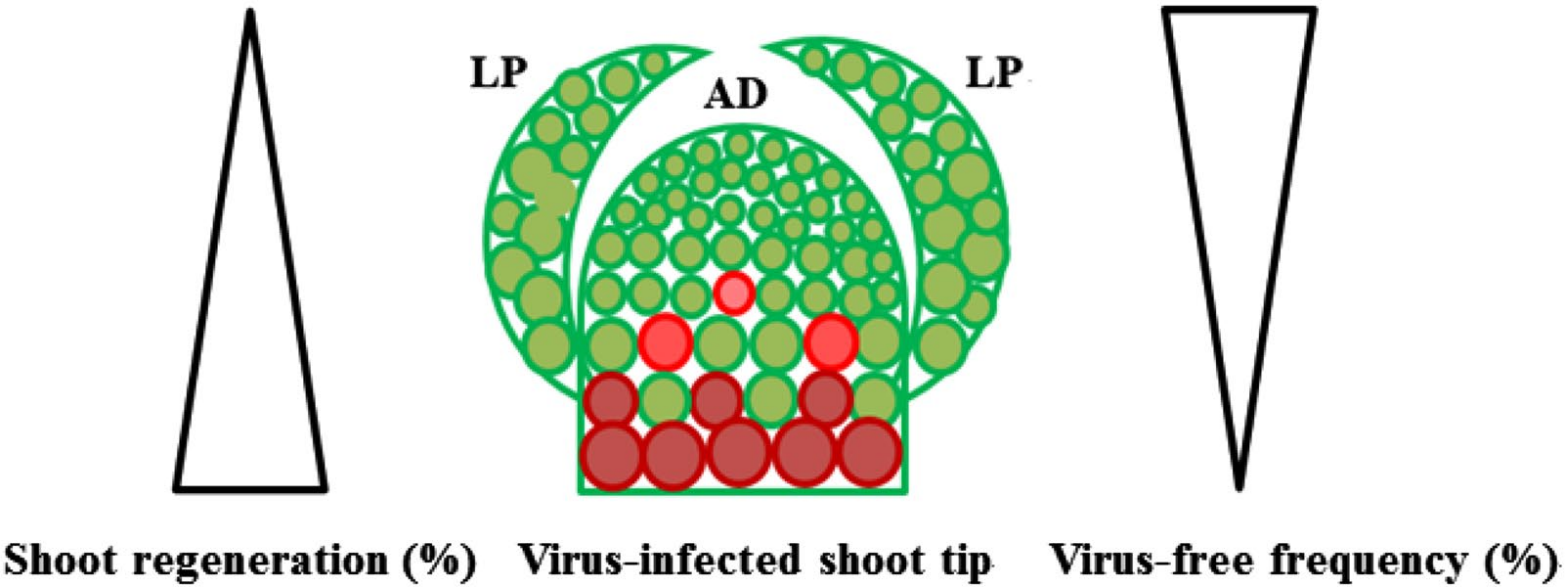
Size of shoot tip in relation with shoot regeneration and virus-free frequency in thermotherapy-based methods. Cells in green color are healthy cells and in red color are virus-infected cells. Increased virus tirer is indicated in increased intensity of red color. *AD* apical dome, *LP* leaf primordium

Shoot tip cryotherapy refers to treatment of infected materials for a short time in liquid nitrogen (LN) using cryopreservation protocols to cure infected plants [24, 25]. When shoot tips are frozen in LN, only cells in the upper parts of the apical dome (AD) are able to survive, while those in the lower parts are killed [26, 27, 47–49]. Virus is unevenly distributed inside plants [50]: virus concentration increases with increased distance from the AD; the AD contains low virus and even free of virus infection (Fig. 2). Thus, plants regenerated from shoot tip cryotherapy may be free of virus infection [24, 25]. Shoot tip cryotherapy has proven to be much more efficient, than the traditional methods like shoot tip culture, for eradication of viruses that do not infect the meristematic cells of the shoot tips [24, 25]. However, shoot tip cryotherapy cannot eradicate the viruses that can infect the meristematic cells of shoot tips, like RBDV [26], ASGV [27, 49], pelargonium flower break virus (PFBV) [51] and pelargonium line pattern virus (PLPV) [51].

Wang et al. [24] reported combining thermotherapy with shoot tip cryotherapy for RBDV eradication. In vitro RBDV-infected shoots of raspberry (*Rubus idaeus*) were thermo-treated using an alternating temperature of 38 °C/26 °C (day/night) under a 16-h photoperiod. After 28–35 days of thermotherapy, shoot tips (0.2 mm in size) containing two leaf primordia (PLs) were excised from the treated shoots and used for cryotherapy, as described by Wang et al. [47]. This procedure produced 20–36% and 30–40% of survival and shoot regrowth levels in cryo-treated shoot tips. About 30–35% plants recovered from combining thermotherapy with shoot cryotherapy were free of RBDV [24].

Recently, combining thermotherapy with shoot tip cryotherapy was shown to efficiently eradicate ASGV, a difficult-to-eradicate virus [27]. In vitro shoots infected with ASGV were thermo-treated using an alternating temperature of 36 °C/32 °C (day/night). After 4 weeks of thermotherapy, shoot tips (1.5 mm in size) containing 4–5 LPs were excised from the treated shoots and subjected to cryotherapy, as described by Li et al. [49]. This protocol yielded 33–76% and 30–100% of shoot regrowth rates and virus eradication frequencies across the four apple cultivars and one rootstock tested. Zhao et al. [27] believed thermotherapy followed by shoot tip cryotherapy might be considered to be the most efficient method so far reported for virus eradication. Some examples of virus eradication by combining thermotherapy with shoot tip cryotherapy are listed in Table 3.

## Key factors affecting success of virus eradication

### Thermotherapy temperatures and durations

High temperatures induce stress to plants, and such stress is intensified as their durations increase [52, 53]. High temperatures and their prolonged durations were shown to reduce survival levels of the treated shoots and shoot tips excised from the treated shoots, the regenerative ability and micrografting success of shoot tips excised from the treated shoots. However, increased temperatures and thermotherapy durations enhanced virus eradication [26, 27, 36, 53].

Applying combining chemotherapy with thermotherapy for virus eradication from apple, Hu et al. [41] found survival levels of the treated in vitro shoots and shoot tips excised from the treated shoots decreased from 100 to 40% and 70 to 8%, respectively, as thermotherapy temperatures increased from 34 to 38 °C. Chemotherapy followed by thermotherapy at 36 °C produced higher frequencies of plants free of ACLSV, ASPV and ASGV than 34 °C [36]. Similar results were repeatedly found in combining chemotherapy with thermotherapy in potato [54], garlic [55] and lily [56], in thermotherapy followed by shoot tip culture in garlic [57], horseradish [53], cassava [54, 55], nectarine [56], plum [57] and pear [58], in combining thermotherapy with shoot tip cryotherapy in raspberry [26] and apple [27], and in thermotherapy followed by micrografting in *Citrus* [41, 42].

Working on combining thermotherapy with shoot tip culture for virus eradication from pear, Tan et al. [63] found that survival levels, shoot length and proliferation efficiency of in vitro shoots decreased, while virus-free frequencies of ACLSV and ASPV increased, as thermotherapy durations increased from 10 to 50 days. Negative effects of prolonged thermotherapy durations on in vitro shoots and shoot tips excised from the treated shoots were frequently found in woody plants such as apricot [64], peach [64], cherry [64], pear [65, 66], plum [62] and black raspberry [67], and herbaceous species such as horseradish [58], chrysanthemum [33], begonia [63] and potato [64]. Increased virus eradication frequencies by increasing thermotherapy durations were also frequently reported in various virus-host combinations. Examples of herbaceous crops included horseradish infected with horseradish mosaic virus (HMV) [53], begonia infected with prunus necrotic ringspot virus (PNRSV) [63], chrysanthemum infected with chrysanthemum virus B (CVB) [28] and potato infected with PVY [64]. Examples of woody plants were apricot infected with ACLSV [59], peach infected with PNRSV and ACLSV [59], sour cherry infected with prune dwarf virus (PDV) and ACLSV [59], raspberry infected with RBDV [22], pear mix-infected with ACLSV, ASPV and ASGV [58] and ACLSV and ASGV [60, 61], and apple infected with ASGV [23].

Preculture of the virus-infected in vitro shoots on a medium containing 10^−5^ M salicylic acid (SA) for 4 weeks increased survival levels of the heat-treated shoots (42 °C, 30 days) from 58 to 64% and PVX-free frequencies from 75 to 98% among the seven potato genotypes [65]. Similar results were also reported by Fang et al. [49] and Aguilar-Camacho et al. [66], who applied thermotherapy-based methods for virus eradication from in vitro potato shoots. SA treatments decreased catalase activity and increased hydrogen peroxide (H_2_O_2_) levels of the in vitro potato shoots, thus enhancing their tolerance to thermotherapy [65, 66]. SA induced plant defense to virus infection and was beneficial for virus eradication [67]. Therefore, SA had double positive effects in thermotherapy for virus eradication: enhancing plant tolerance to thermotherapy and increasing virus eradication frequency. Detailed information of SA-mediated biotic and abiotic stress signalling in plants can be found in a recent review [67].

### Step-wise increasing temperatures and alternating temperatures

Many temperate plant species like *Prunus*, *Vaccinium* and *Vitis* are sensitive to sudden increased temperatures, and step-wise increasing temperature treatments (preconditioning) helped them adapt themselves to thermotherapy [68]. In the study of Bruna [52] for eradication of onion yellow dwarf virus (OYDV), in vitro shoots of garlic were preconditioned at 30 °C for 7 days and then heat-treated at 38 °C for 38 days. Step-wise increasing temperatures were also used in *Prunus* fruits such as peach [68], nectarine [56] and apricot [69].

Thermotherapy using an alternating day/night temperature was found to alleviate negative effects of high constant temperature on the in vitro shoots during thermotherapy and increased survival and growth of the heat-treated shoots, thus improving virus eradication efficiency. For example, Knapp et al. [14] reported that shoot survival levels were much higher in in vitro apple shoots that had been heat-treated for 30 days by an alternating temperature of 38 °C/36 °C (day/night) than by a constant temperature of 38 °C. Higher survival levels, and greater shoot length and proliferation index were obtained in in vitro pear shoots that had been heat-treated for 50 days by an alternating temperature of 42 °C/34 °C (day/night) than by a consistent temperature of 37 °C [58]. Beneficial effects of the alternating temperature for thermotherapy on the treated shoots were also observed in other woody plants like peach [68], pear [70], apple [23, 71], plum [57] and grapevine [24].

### Types and concentrations of antivirus agents

Types and concentrations of antivirus agents used in thermotherapy-based chemotherapy influenced survival of the treated in vitro shoots, shoot regeneration of shoot tips excised from the treated shoots and virus eradication frequencies. Verma et al. [68] tested the effects of three antivirus agents (6-azauracil, 2-thiouracil and ribavirin) on PNRSV eradication from the infected in vitro Begonia shoots, and found chemotherapy of 20–35 mg L^−1^ ribavirin yielded 20–45% of virus-free plants, which were higher than 0–15% and 0–20% produced by the same concentrations of 2-thiouracil and 6-azauracil, respectively. Chemotherapy (20 mg L^−1^ ribavirin) followed by thermotherapy (38 °C, 25 days) yielded almost 100% of shoot survival levels and 57.5% of PNRSV-free plants. Rribavirin treatment (30 mg L^−1^) followed by thermotherapy (38 °C, 25 days) produced less than 30% shoot survival and 75% of PNRSV-free plants. Reduced survival levels of the treated shoots by increased ribavirin concentrations were reported in garlic [16], pear [31, 76], apple [44, 75], raspberry [76] and grapevine [77] for grapevine. Optimal ribavirin concentration for production of virus-free plants was 12.21 mg L^−1^ (50 μM used) for lily infected with lily symptomless virus (LSV), tulip breaking virus-lily (TBV-L) and cucumber mosaic virus (CMV) [7], 20 mg L^−1^ for potato infected with PLRV and PVY [49, 74], *Begonia* infected with PNRSV [68], artichoke infected with artichoke latent virus (ALV) [32] and cassava infected with east African cassava mosaic virus (EACMV), [60], 25 mg L^−1^ for apple infected with ACLSV, ASPV and ASGV [36] and pear infected with ACLSV and ASGV [65], 50 mg L^−1^ for pear infected with ACLSV [31], garlic infected with OYDV, leek yellow stripe virus (LYSV), shallot latent virus (SLV), mite borne filamentous virus (MbFV) and garlic common latent virus (GCLV) [16].

Ram et al. [33] found chemotherapy (30–40 mg L^−1^ 2-thiouracil) followed by thermotherapy (38 °C, 30 days) was most effective in CVB eradication among the five antiviral chemicals tested. 2-thiouracil was also shown to be effective in eradicating LSV, TBV-L and CMV from lily [73], and PLRV from potato [34].

Filter-sterilized antivirus agents caused much severer toxic to plants but produced higher frequencies of PVY-free plants than the autoclaved agents, indicating autoclaving may reduce effects of antivirus agents on virus eradication [79]. In many cases, filter-sterilized antivirus agents were added to the medium after autoclaving [34, 55, 60, 69, 77, 80, 81], and in some cases, antivirus agents were added to the medium before autoclaving [35].

### Size of shoot tips

As addressed above, virus is unevenly distributed inside plants [50] (Fig. 2). Therefore, size of shoot tips is critical for virus eradication. In general, size of shoot tips is positively related to survival and shoot regeneration, while it is negatively proportional to virus eradication frequency [21], (Fig. 2). With grapevine, Skiada et al. [28] reported although shoot regeneration levels were much higher (80%) in shoot tip culture (0.5 cm) than that (56%) in meristem culture (0.1–0.2 mm), the latter produced 91.2% and 73.8% plants free of GLRaV-1 and GRSPaV-1, which were much higher than 68% and 51% by the former. Similar results were obtained in a great number of studies using thermotherapy-based methods for eradication of different types of viruses from various plant species ranging from woody to herbaceous plants and originating from temperate to tropical regions [2, 12, 19, 21, 23]. Shoot tip size is critical for virus eradication and combining thermotherapy with shoot tip culture allows use of larger shoot tips than those used for shoot tip culture without thermotherapy [12, 19, 21, 23].

### Source of explants and position of shoot tips

Source of explants and shoot tip position influenced success of virus eradication in thermotherapy-based methods. Survival and growth levels of apple shoot tips following thermotherapy were higher in the buds harvested from the actively growing shoots than from the dormant ones, and in the apical buds than in axillary ones [82]. These effects were related to higher endogenous contents of auxins and cytokinins in the actively growing and apical buds than the dormant and axillary ones [83]. Survival levels of the heat-treated grapevine shoots were higher when in vitro cultures were established from the middle and basal buds than from the terminal buds [84]. GLRaV-3-free frequencies were similar among the terminal, the first and second axillary shoot tips, but were lower in the third axillary shoot tips [84]. Virus-free frequencies were higher plants when the diseased in vitro shoots were established in summer than in spring [84]. The authors attributed this effect to “natural thermotherapy’’, since when the samples were collected in summer, the day temperatures in the vineyards (Murcia, Spain) reached 38–40 °C [84]. Hu et al. [36] found virus elimination frequencies were higher in the plants regenerated from axillary shoots than from apical shoots in apple, using combining chemotherapy with thermotherapy. The authors believed the axillary shoot tips elongated faster than apical ones in their experiments, thus helping new growth of the axillar buds to escape virus infection [36].

### Genotype-specific responses

Survival levels of the heat-treated shoots, and survival and shoot regrowth levels of the shoots tips excised from the heat-treated shoots of *Capparis spinosa* were significantly higher in ‘Pantelleria’ than in ‘Salina’ [85]. Similar virus-free frequencies were obtained between these two cultivars by combining thermotherapy with shoot tip culture. Survival levels of the heat-treated shoots and virus free frequencies differed from two apple cultivars by thermotherapy followed by shoot tip culture [75]. Genotype-specific responses were also found in a number of plants including woody species such as *Prunus* [64] and *Vitis* [86], and herbaceous species such as *Allium sativum* [55, 87], artichoke infected with ALV [32], *Solanum tuberosum* [29], *Lilium* [56, 88], *Cynara cardunculus* var. *scolymus* [89] and *Manihot esculenta* [59].

It has been known that tolerance/resistance to high temperatures varies with plant genotypes [53]. Ability of a given virus to infect meristematic cells of the shoot tips and its distribution pattern in shoot tips following heat treatment also vary with plant genotypes [6, 27, 90]. These variations lead to genotype-specific responses to thermotherapy-based methods for success of virus eradication. For example, using in situ hybridization for localization of chrysanthemum stunt viroid (CSVd) in shoot tips of the four infected *Argyranthemum* genotypes, Zhang et al. [90] reported CSVd was present in all tissues including the uppermost cell layers in the apical dome (AD) and the youngest leaf primordia (LPs) 1 and 2 in ‘Yellow Empire’ and ‘Butterfly’, but it was detected only in the lower part of the AD, while not in the upper part of the AD, and LPs 1–2 in ‘Border Dark Red’ and ‘Border’. Virus distribution patterns analyzed by immunohistologial localization were similar in shoot tips of the ASGV-infected apple cultivars ‘Gala’ and ‘Ruixue’ before thermotherapy [27]. However, 4 weeks of thermotherapy resulted in different virus distribution patterns between them [27]. In ‘Gala’, the virus was not detected in the AD and LPs 1–5 but was detected in LP 6 and older LPs of the shoot tips. In ‘Ruixue’, the virus was not detected in the AD and LPs 1–3 but was found in LP 4 and older LPs [27].

### Types of virus and infection status

In general, phloem-limited viruses are easy to eradicate, while those that can infect the meristematic cells of shoot tips are difficult to eradicate.

*Artichoke latent virus* (ArLV) could be easily eliminated by shoot tip culture alone, while *artichoke Italian latent virus* (AILV) could be removed only when thermotherapy followed by shoot tip culture was used [89]. Virus-free frequencies were much higher for GLRaV-1 (67–91%) than for GRSPaV-1 (51–74%), regardless of size of shoot tips used in combining thermotherapy with shoot tip culture [28]. Thermotherapy followed by shoot tip culture resulted in virus-free frequencies of 100% for grapevine fanleaf virus (GFLV), 70% for grapevine virus A (GVA), 25 for GLRaV-1, 25 for GLRaV-3 and 0% for grapevine fleck virus (GFKV) [91]. Varying virus-free frequencies with types of the virus were repeatedly reported in various virus-host combinations by thermotherapy-based methods, for example, garlic infected with LYSV, OYDV, GarMbFV and GCLV [87], potato infected with PVY and PLRV [49]; apple infected with ACLSV, ApMV, ASPV and ASGV [36, 75, 82], pear infected with ACLSV and ASGV [65, 66]; figs infected with fig leaf mottle-associated virus 1 (FLMaV-1), FLMaV-2 and fig mosaic virus (FMV) [92].

Viruses differ in their abilities to infect shoot tips of the same plant [6], thus leading to differences in virus eradication frequencies in different viruses from the same plant. Wang and Valkonen [48] found sweetpotato chlorotic stunt virus (SPCSV) and sweetpotato feathery mottle virus (SPFMV) were not present in the AD and LPs 1–3 of the shoot tips of sweetpotato. SPCSV was detected in LP5 and the older tissue, but not in LP 1–4, and SPFMV in LP4 and the older tissue. Li et al. [49] found that ASPV was present in lower part of AD, and LP 4 and older tissues of the shoot tips, but not present in upper part of AD and LPs 1–3, leaving a ASPV-free area in AD (approximately 0.8 mm in length). ASGV was detected across the AD and LPs 1–6 of the shoot tips, leaving only the very top layers of cells in AD (approximately 0.5 mm in length) free of ASGV infection. Similar distribution patterns of ASPV and ASGV were observed in shoot tips of apple ‘Gala’ [93]. These data provided explanations to why virus-free frequencies varied with the types of virus in thermotherapy-based methods.

Virus eradication was easier from single-infected plants than from mix-infected ones [12]. Following combining thermotherapy with shoot tip culture, Knapp et al. [18] found ACLSV-free frequency was much higher in the in vitro single-infected shoots than in the shoots mix-infected with ASGV and ACLSV. Fang et al. [54] also reported PLRV was much easier to remove from single-infected shoots than co-infected ones with PVY and PLVR. However, little has been known about mechanism of the synergistic effects of viruses in terms of virus eradication.

## Genetic stability and field behaviors in virus-free plants

High temperatures cause stress to plants [52, 53] and antivirus chemicals are toxic to plants [19, 31, 34, 68, 80, 81]. These create ricks of genetic variations in plants regenerated following thermotherapy-based methods. In addition, in vitro culture procedure may also induce genetic variations, due to the use of high concentrations of plant growth regulators, repeated subculture and shoot regeneration through callus formation [94, 95]. The purpose of cultivation of virus-free plants is to improve quality and yield of agricultural production, while maintaining the genetic stability and unique straits of the original cultivars [1, 6]. Therefore, it is necessary to assess genetic stability and observe field behaviors in virus-free plants derived from thermotherapy-based methods. Unfortunately, information on the said subject has been quite limited.

Acquadro et al. [89] applied simple sequence repeat (SSR) and amplified fragment length polymorphism (AFLP) markers to assess genetic stability in virus-free Globe artichoke plants obtained by thermotherapy followed by shoot tip culture. SSR did not detect any polymorphic bands. Although some polymorphic bands were detected by AFLP, no obvious variations were found between the control and treated samples, and polymorphic bands detected were genotype-specific. Nevertheless, these results indicate thermotherapy may induce genetic variations.

Nesi et al. [88] reported LSV-free lily plants produced by combining thermotherapy with shoot tip culture grew more vigorously and produced more leaves than the virus-infected plants after the first year of growth in the field. Ram et al. [96] observed field behaviors of CMV, CVB and TAV-free chrysanthemum plants produced by thermotherapy followed by shoot tip culture, and found the virus-free plants produced significantly higher vegetative growth (plant height and number of stems) and flower production (number and diameter of flowers) than the infected plants. Ramírez-Malagón et al. [55] reported *Potyvirus*-free garlic plants derived from thermotherapy or chemotherapy followed by shoot tip culture had better vegetative growth and higher yield and quality of cloves than the virus-infected plants. Compared field behaviors between virus-infected potato plants and virus-free plants derived from virus-free minitubers resulted from thermotherapy followed by shoot tip culture, Mahmound et al. [69] found plant height, and tuber weight and yield were significantly greater in virus-free plants than those single-infected with PVY, and mix-infected with PLRV, PVX and PVY. These data indicated virus-free plants produced by thermotherapy-based methods significantly increased their potential of vegetative growth and yield, compared with virus-infected ones.

## Mechanism

### Thermotherapy

Possible mechanisms involved in thermotherapy-based methods for virus eradication were well discussed in a number of publications [12, 19, 22, 23]. Recent studies added valuable information on the said subject:

1. High temperature treatments were found to prevent virus movement toward the meristematic cells of the treated shoots of raspberry infected with RBDV [26], pear infected with ACLSV and ASGV [63], and apple infected with ACLSV and ASGV [27]. This effect resulted in production of larger virus-free areas of the infected shoot tips, thus helping virus eradication [26, 27].
2. Thermotherapy was found to inhibit viral replication [92, 97] or caused virus RNA degradation [26, 27], thus decreasing virus titer in the infected shoot tips. Liu et al. [93] found reduced ASGV titers in thermo-treated pear shoot tips were associated with a number of miRNA-mediated genes related to disease defense and hormone signal transduction pathways in the apical meristem of pear shoots. These results suggested that miRNAs may have important functions in the thermotherapy-induced decreases of virus titer in the heat-treated shoots [98].
3. High temperatures promoted virus-induced RNA silencing in tobacco infected with cymbidium *ringspot virus* (CRSV) [99] and PVX [100], cassava and tobacco infected with cassava *Geminivirus* [101] and raspberry infected with RBDV [26]. Recently, Liu et al. [97] found thermotherapy drastically decreased viral genome accumulation in shoot tips of the treated pear shoots infected with ASGV, which was accompanied with the elevated levels of virus-derived small interfering RNA (vsiRNA). Thermotherapy induced the biogenesis of vsiRNAs and inhibited viral RNA accumulation, by up-regulating the expression of key genes in the RNA silencing pathway [102]. miRNAs have been known to regulate key genes in resistance to virus infections [103–106]. Increased virus-induced silencing by thermotherapy is so far the most convinced mechanism involved in improved virus eradication by thermotherapy-based methods [26, 98, 102]. Nevertheless, specific mechanism as to why thermotherapy improves virus eradication has not been well understood yet and needs further studies.

### Chemotherapy

Studies that elucidate mechanism involved in antivirus agents for plant virus have been quite limited. Existing studies demonstrated exogenous applications of antivirus agents inhibited synthesis of virus RNA [107–113]. Inhibition of viral RNA synthesis decreased the number of virus particles released from the infected cells into the newly divided cells [107, 108], thus resulting in production of a larger virus-free area or reduction of virus titers in the treated shoot tips and helping virus eradication [34]. Applying ribavirin to treat human cells infected with hepatitis C virus, a RNA virus, Crotty et al. [114, 115] found the antiviral activity of ribavirin was through lethal mutagenic activity by forcing the virus into ‘error catastrophe’, thus destroying the infectivity of the virus genomic RNA. Nevertheless, working model of chemotherapy on plant virus eradication has not been well understood yet and extra studies are needed.

### Cryotherapy

As addressed above, distribution of virus is uneven inside plants [50]. When shoot tips are frozen in LN, only cells that are less differentiated, have large nucleo-cytoplasmic ratio and contain less free water are able to survive [24, 25]. Therefore, only cells in top layers of AD and the youngest leaf primordia (LPs) are able to survive, while cells in lower parts of AD and the older PLs are killed, following cryopreservation [24, 25]. Thus, freezing in LN kills virus-infected cells and allows healthy (virus-free) cells to survive and then regenerate into pathogen-free plants [20–22]. In combining thermotherapy with cryotherapy, thermotherapy had dual effects: production of larger virus-free areas in the treated shoots, as addressed above, and reduction in the number of survival cells, eventually improving virus eradication frequency, compared with cryotherapy alone [26, 27].

## Further prospects

So far, various thermotherapy-based methods have successfully been developed for eradication of viruses from almost all economically important crops that are vegetatively propagated [2, 12, 19, 22, 23], Tables 1, 2, and 3 in present study. However, simple and efficient methods are still needed for eradication of the viruses, especially those that infect meristematic cells of the shoot tips. RBDV and ASGV are the two representatives of this type of virus and can be effectively eradicated only by combining thermotherapy with shoot tip cryotherapy [25, 26]. Compared with other techniques, cryotherapy is a relatively new technique, and may not be available in many laboratories, which is still limiting wider applications of this method. Therefore, continuous developments of simple and efficient method for eradication of such viruses are necessary.

Methods of virus detection and durations after virus eradication treatments determine virus eradication frequencies in a given method. In many of the previous studies, ELISA-based methods were used to screen the virus status, followed by PCR-based methods to confirm the virus status in plants following virus eradication treatments [17, 34, 42, 116]. In some cases, only ELISA-based methods were used to confirm the virus status in the plants following virus eradication [29, 30, 55, 61, 65, 71, 78]. It is well-known that ELISA-based methods are much less sensitive to detect viruses than PCR-based methods [2, 117]. Often, virus-free frequencies analyzed by ELISA-based methods in plants following virus eradication treatments were much higher than by PCR-based ones [28, 36, 56, 63, 72]. Reduction of virus titer, but not virus eradication, was frequently found in the plants following virus eradication treatments [51, 119]. In such cases, ELISA-based methods or too short time durations after virus eradication treatments failed to detect the ‘real’ virus status in the plants. Using combining thermotherapy with chemotherapy, Xu et al. [78] reported virus was detected by ELISA only in 30% and 6% of in vitro regenerants of lily ‘Georgia’ and ‘Casablanca’, respectively. However, the virus infection analyzed by the same method increased to 100% and 44% in these two cultivars after 6 months of growth in soil under greenhouse condition. Based on these data, we support use of two unrelated methods, for example, combining PCR-based methods with ELISA or biological indexing, or even more sensitive methods like next generation sequencing [2, 117] for detection of virus status in plants following virus eradication treatments, thus increasing the reliability of the results. In addition, to ensure virus status in the regenerants following virus eradication treatments, double tests for viruses should be done: first in in vitro regenerants following virus eradication treatments and then in plants after at least 6 and 10 months (including a dormant season) of growth in soil for herbaceous and woody plants, respectively [16, 27, 49, 56].

Many of the previous studies used single cultivar or genotype for virus eradication. As addressed above, genotype-specific response is very common in virus eradication studies, and a protocol may work well in a given genotype but completely fails in another. Therefore, development of protocols applicable to a wide range of genotypes within a species would facilitate wider applications of the technique to production of virus-free plants for commercial cultivation and preservation of plant germplasm [16, 17, 27].

Extra studies should also be strengthened in assessments of genetic stability and observations on field performance in the virus-free plants resulted from thermotherapy-based methods. These studies would accelerate extensions of virus-free plants to practical agricultural production for improvements of yield and quality of crops.

## Abbreviations

ACLSV: apple chlorotic leaf spot virus
AD: apical dome
AILV: artichoke Italian latent virus
ALV or ArLV: artichoke latent virus
ASGV: apple stem grooving virus
ASPV: apple stem pitting virus
AVA: arracacha virus A
AVB: arracacha virus B
CMV: cucumber mosaic virus
CSVd: chrysanthemum stunt viroid
CTLV: citrus tatter leaf virus
CTV: citrus tristeza virus
CVB: chrysanthemum virus B
SDV: satsuma dwarf virus
EACMV: East African cassava mosaic virus
EU: European Union
FLMaV: fig leaf mottle-associated virus
FMV: fig mosaic virus
GCLV: garlic common latent virus
GFKV: grapevine fleck maculavirus
GFLV: grapevine fanleaf nepovirus
GLRaV-1: grapevine leafroll-associated virus 1
GRSPaV: grapevine rupestris stem pitting-associated virus
GVA: grapevine vitivirus A
HMV: horseradish mosaic virus
ICRSV: Indian citrus ringspot virus
MbFV: mite borne filamentous viruses
LPs: leaf primordia
LSV: lily symptomless virus
LN: liquid nitrogen
LYSV: leek yellow stripe virus
OYDV: onion yellow dwarf virus
PapMV: papaya mosaic virus
PDV: prune dwarf virus
PFBV: pelargonium flower break virus
PLRV: potato leafroll virus
PLPV: pelargonium line pattern virus
PNRSV: prunus necrotic ringspot virus
PPV: plum pox virus
PVS: potato virus S
PVX: potato virus X
PVY: potato virus Y
RBDV: raspberry bushy dwarf virus
SA: salicylic acid
SLV: shallot latent virus
SPCSV: sweetpotato chlorotic stunt virus
SPFMV: sweetpotato feathery mottle virus
SSR: simple sequence repeat
AFLP: amplified fragment length polymorphism
TAV: tomato aspermy virus
TBV-L: tulip breaking virus-lily
UMMV: ullucus mild mottle virus
UMV: ullucus mosaic virus
UVC: ullucus virus C
vsiRNA: virus-derived small interfering RNA
